# On Gaussian curvature and membrane fission

**DOI:** 10.1038/s41598-021-88851-y

**Published:** 2021-05-05

**Authors:** Mara Denisse Rueda-Contreras, Andreu F. Gallen, J. Roberto Romero-Arias, Aurora Hernandez-Machado, Rafael A. Barrio

**Affiliations:** 1 Instituto de Física, U.N.A.M., Ap. Postal 101000, 01000 Mexico, DF Mexico; 2 Departament Física de la Matèria Condensada, Universitat de Barcelona, 08028 Barcelona, Spain; 3 Instituto de Investigaciones en Matemáticas Aplicadas y en Sistemas, Universidad Nacional Autónoma de México, 01000 Mexico, Mexico; 4 Institute of Nanoscience and Nanotechnology (IN2UB), 08028 Barcelona, Spain

**Keywords:** Biological physics, Phase transitions and critical phenomena, Nonlinear phenomena, Applied mathematics, Membrane biophysics, Membrane structure and assembly

## Abstract

We propose a three-dimensional mathematical model to describe dynamical processes of membrane fission. The model is based on a phase field equation that includes the Gaussian curvature contribution to the bending energy. With the addition of the Gaussian curvature energy term numerical simulations agree with the predictions that tubular shapes can break down into multiple vesicles. A dispersion relation obtained with linear analysis predicts the wavelength of the instability and the number of formed vesicles. Finally, a membrane shape diagram is obtained for the different Gaussian and bending modulus, showing different shape regimes.

## Introduction

Vesicle formation is a fundamental process in many biological systems, e.g., the Golgi apparatus^1,2^, the synaptic system^3,4^, or enveloped viruses^5,6^. The Golgi apparatus constantly releases transport vesicles filled with proteins that are carried to other parts of the cell. In the synaptic nerve terminals, vesicles are filled with neurotransmitters and released by exocytosis^3^. Moreover, many viruses are enveloped by a lipid membrane which mediates the fusion of the virus with the host cell membrane; some examples are HIV-1, herpesviruses, the Ebola virus^5^, and coronaviruses^7^ like SARS-CoV-2.

There is a fair amount of research on cellular membrane deformation and morphology^8,9^. However, there is little understanding when topological transitions occur and Gaussian curvature plays a role^8–10^. The Gauss-Bonnet theorem states that the integral of the Gaussian curvature over a surface is proportional to the surface Euler characteristic^11^. This assures that the Gaussian curvature term is topologically invariant. This leads to the term being ignored for homogeneous systems. However, it is fundamental for topological transitions like fusion or fission. Lipid bilayers exhibit different stable configurations, depending on the values of the Gaussian and bending energetic moduli. There are no direct experimental measurements of the Gaussian modulus, although a method has been proposed recently^12^. Indirect measurements give a negative value of about $\bar{\kappa }' \approx -15 K_BT$^13^, and molecular dynamics simulations give similar results^14^. The negative sign implies that the energetic term of the Gaussian curvature favors fission, since fission increases the Euler characteristic. The most common fission event is the formation of a vesicle.

In all cases what seems a necessary requirement for fission is a large membrane curvature on the area where a vesicle is to be generated, which can be modeled with a spontaneous curvature. The final fission of the vesicle is often mediated by very specific proteins, although this is not always the case. Large spontaneous curvature can suffice to produce fission^15–17^. This can be accomplished by interactions among membrane-bound proteins^15^ or by a osmotically induced pearling instability^16^.

There has been extensive research for protein mediated fission. The dynamin superfamily^8,18–20^ or the ESCRT machinery^5,21,22^ are two different sets of proteins that mediate in budding and fission. For example, the dynamin Drp1 is considered a major component in mitochondrial division; other dynamics are considered to either help or be necessary for the fission process. Previous numerical work^23,24^ on budding or fission at mesoscopic scales are based on the evolution of the membrane up to the instant prior to fission. Pearling of tubes has also been studied with the inclusion of the Gaussian energy^25,26^, and some regimes where the Gaussian modulus makes the tube topologically stable are studied in those works. In some instances, on the other hand, the neck which connects the vesicles being formed may be stabilized by lateral segregation of membrane components^27–31^.

We have developed a three-dimensional model to study the dynamical evolution of a membrane, including not only the mean curvature energy term but also the Gaussian energy term. This is done by a phase-field methodology, which has been used to study a variety of systems based on the Helfrich theory for cellular membranes^32–41^. Our description is mesoscopic considering a two dimensional diffuse interfase, in contrast to the microscopic view, which describes the rearrangement of bilayers^13^. The phase field approach allows to study not only the equilibrium shapes, but also the dynamics of the formation of vesicles, in the same spirit as the Landau-Ginzburg framework.

Numerical integration results show that without the inclusion of the Gaussian curvature the system exhibits a pearling instability, which has been already observed in many simulations^33,42,43^ and experiments^44,45^. Pearling happens in membranes due to the spontaneous curvature, and the Gaussian curvature may lead to the fission of the pearls. Our phase field model explores the fission events where the Gaussian curvature is relevant, and the results compare extremely well with the ones observed in experiments.

## Methods

### The model

Phase-field approaches are suitable to model the dynamics of membranes that change their shape under certain conditions^32–40^. As the Gaussian curvature is an intrinsic property of the surfaces, no matter their dimensions or the metric relations that can be exerted within them^46^, it certainly has an influence on the way membranes can change their shape. However, Gaussian curvature has not been considered in phase field models because its contribution to the energy is a topological invariant. Nonetheless, it has to be included in the study of membrane dynamics because the whole curvature energy depends on it and not only on the usual bending rigidity modulus due to the mean curvature. Minimization of the entire curvature energy allows to describe, not only the shape changes that membranes must acquire, but some processes that involve a change of genus, such as fission and fusion.

Starting off from the expression for the bending energy due the mean curvature *H* and the Gaussian curvature *K* we have

1 $$\begin{aligned} {\mathscr {F}} = \frac{ \bar{\kappa }}{2}\int _{\Gamma } (2H-c_0)^2 ds + \bar{\kappa }' \int _{\Gamma } K ds, \end{aligned}$$

where $\bar{\kappa }$ and $\bar{\kappa }'$ are the bending modulus and Gaussian modulus, $c_0$ is the spontaneous curvature and the integral is calculated over the whole membrane surface $\Gamma$. In here we include the influence of the Gaussian curvature *K* in the dynamics of the system in order to model situations in which the genus of the membrane changes.

In terms of the principal curvatures of the surface, the free energy of Eq. (1) can be written as follows:

2 $$\begin{aligned} {\mathscr {F}}&= \frac{\bar{\kappa }}{2}\int _{\Gamma } \left( \frac{1}{R_1}+\frac{1}{R_2}-c_0\right) ^2 ds + \bar{\kappa }' \int _{\Gamma } \left( \frac{1}{R_1R_2}\right) ds, \end{aligned}$$

where $R_i$ are the principal radii of curvature. Note that the mean curvature has dimensions of inverse length, and the Gaussian curvature has dimensions of inverse length squared.

A phase-field model of the Cahn–Hilliard type can be defined from Eq. (1), as in Ref.^32^, in which the authors express the free energy ${\mathscr {F}}$ of the system as an expansion of powers of a smooth scalar field $\phi :\Omega \subset {\mathbf {R^3}} \longrightarrow {\mathbf {R}}$, that acts as an order parameter:

3 $$\begin{aligned} {\mathscr {F}} = \int _{\Omega } {\mathscr {L}}(\phi , \nabla \phi , \nabla ^2 \phi ) dV. \end{aligned}$$

Assuming that the system is isotropic and homogeneous, and given that the order parameter must have two stable phases, the energy density ${\mathscr {L}}$ can be written as ${\mathscr {L}}=\left( \Phi [\phi ] \right) ^2$, where the functional is defined as $\Phi [\phi ] = -\phi + \phi ^3 - \varepsilon ^2 \nabla ^2 \phi .$

The parameter $\varepsilon$ represents the width of the interface between the two phases. One of the stable phases, typically defined by $\phi =1$, corresponds to the interior of the volume delimited by the membrane located at $\phi =0$, whereas $\phi =-1$ represents the outer environment.

It can be demonstrated that Eq. (3) with ${\mathscr {L}}=\left( \Phi [\phi ] \right) ^2$ is equivalent to the expression of the bending energy of the surface in terms of the mean curvature *H*^32^. This would model the first half of Eq. (1), only the Gaussian term remains to be portrayed in a phase field approach.

The Gaussian curvature term can be defined in terms of the curvature tensor $Q_{\alpha \beta }$ of the surface as

4 $$\begin{aligned} K = \sum _{\alpha ,\beta } \left[ \left( Q_{\alpha \alpha }Q_{\beta \beta }-Q_{\alpha \beta }^2 \right) \frac{1-\delta _{\alpha \beta }}{2} \right] , \end{aligned}$$

which in turn can be defined in terms of the gradients of the order parameter $\phi$ as^47^,

5 $$\begin{aligned} Q_{\alpha \beta } = \frac{\sqrt{2}\varepsilon }{1-\phi ^2} \left[ \partial _{\alpha \beta } \phi + \frac{2\phi }{1-\phi ^2} \partial _{\alpha } \phi \partial _{\beta } \phi \right] , \end{aligned}$$

where $$\partial _{\alpha }={\partial }/{\partial x_\alpha }$$, and $$\partial _{\alpha \beta } = {\partial ^2}/{\partial x_\alpha \partial x_\beta }$$.

The free energy $F={\mathscr {F_{SC}}}+{\mathscr {F_{G}}}$ of the system is then represented by the spontaneous curvature model

6 $$\begin{aligned} \mathscr {F_{SC}} = \kappa \int _\Omega \left( (\phi -\varepsilon C_0)(\phi ^2-1)- \varepsilon ^2 \nabla ^2 \phi \right) ^2 dV, \end{aligned}$$

where $\kappa = 3\sqrt{2}\bar{\kappa }/16\varepsilon ^3$, $C_0 =c_0/\sqrt{2}$, plus the energy density due to the Gaussian curvature,

7 $$\begin{aligned}&{\mathscr {F_{G}}} = \frac{\kappa '}{2\varepsilon ^2} \int _\Omega \tilde{K} dV = \frac{\kappa '}{2\varepsilon ^2} \int _\Omega K (1-\phi ^2)^2 dV\nonumber \\&\quad = \frac{\kappa '}{2\varepsilon ^2} \int _\Omega \sum _{\alpha <\beta } (1-\phi ^2)^2 \left[ Q_{\alpha \alpha }Q_{\beta \beta }-Q_{\alpha \beta }^2 \right] dV, \end{aligned}$$

where $\kappa ' = 3\sqrt{2}\varepsilon \bar{\kappa }'/4$. Both terms determine the complete bending energy of the system. The time evolution of the phase-field is set according to the Cahn–Hilliard dynamics, since the volume is supposed to be locally conserved^32–37^. The variations of the free energy with respect to $\phi$ must then be subjected to diffusion, yielding a dynamic equation for the phase-field governed by^32^,

8 $$\begin{aligned} \frac{\partial \phi }{\partial t} = \nabla ^2 \left( \frac{\delta {\mathscr {F_{SC}}} }{\delta \phi } + \frac{\delta{\mathscr {F_{G}}} }{\delta \phi } \right) . \end{aligned}$$

We can express this last equation in terms of the order parameter and its spatial variations only (see “Appendix A”). The final result, after some algebra, is,

9 $$\begin{aligned} \frac{\partial \phi }{\partial t} = \kappa \nabla ^2 \left( (3\phi ^2-1-2\phi \varepsilon C_0)\mu -\varepsilon ^2\nabla ^2\mu + \sigma [\phi ]\nabla ^2\phi \right) -\kappa ' \nabla ^2 \left( \frac{12\phi }{1-\phi ^2} F_{K_1} + \frac{2(3\phi ^2+1)}{(1-\phi ^2)^2} F_{K_2} \right) , \end{aligned}$$

where $\mu = (\phi -\varepsilon C_0)(\phi ^2-1)- \varepsilon ^2 \nabla ^2 \phi$. The terms $F_{K_i}$ represent the Gaussian curvature effect and are, explicitly,

$$\begin{aligned} F_{K_1}= \partial _{\alpha \alpha } \phi \partial _{\beta \beta } \phi - (\partial _{\alpha \beta } \phi )^2, \end{aligned}$$

and

$$\begin{aligned} F_{K_2} = \partial _{\alpha \alpha } \phi (\partial _\beta \phi )^2 + \partial _{\beta \beta } \phi (\partial _\alpha \phi )^2 -2 \partial _{\alpha \beta } \phi \partial _{\alpha }\phi \partial _{\beta }\phi . \end{aligned}$$

The parameter $\sigma [\phi ]$ is a Lagrange multiplier that depends on the field $\phi$ and assures area conservation^47^. One can determine $\sigma [\phi ]$ by calculating the area $S \propto \int _\Omega |\nabla \phi |^2dV$ and demanding that $dS/dt\approx 0$. Using that Eq. (8) guarantees the conservation of the field $\phi$, one obtains

10 $$\begin{aligned} \sigma [\phi ] = -\frac{\int _\Omega \nabla \phi \cdot \nabla [\nabla ^2 \big (\frac{\delta \mathscr {F_{SC}} }{\delta \phi } + \frac{\delta \mathscr {F_{G}} }{\delta \phi }\big )]dV }{ \int _\Omega \nabla \phi \cdot \nabla [\nabla ^2(\nabla ^2\phi )] dV}. \end{aligned}$$

## Results

### Numerical simulations

In principle, by solving Eqs. (9–10), it is possible to model situations in which the topological genus of the membrane changes, as in vesicle formation. Thus, we performed three dimensional calculations using the same method of integration as in^32^. We used a finite-difference scheme for the spatial discretization and an Euler method for the temporal derivatives with the appropriate small time step of $dt=10^{-5}$ to ensure enough accuracy and avoid artefacts^48^. For the Gaussian curvature term in Eq. (9) we implement the integration using the residue theorem over the positive complex plane. As the initial membrane, we choose a cylindrical shape of radius *R* and length *L* closed at the top and open at the bottom. Its base is in contact with the wall of the domain and we are using zero flux boundary conditions.

**Figure 1:**
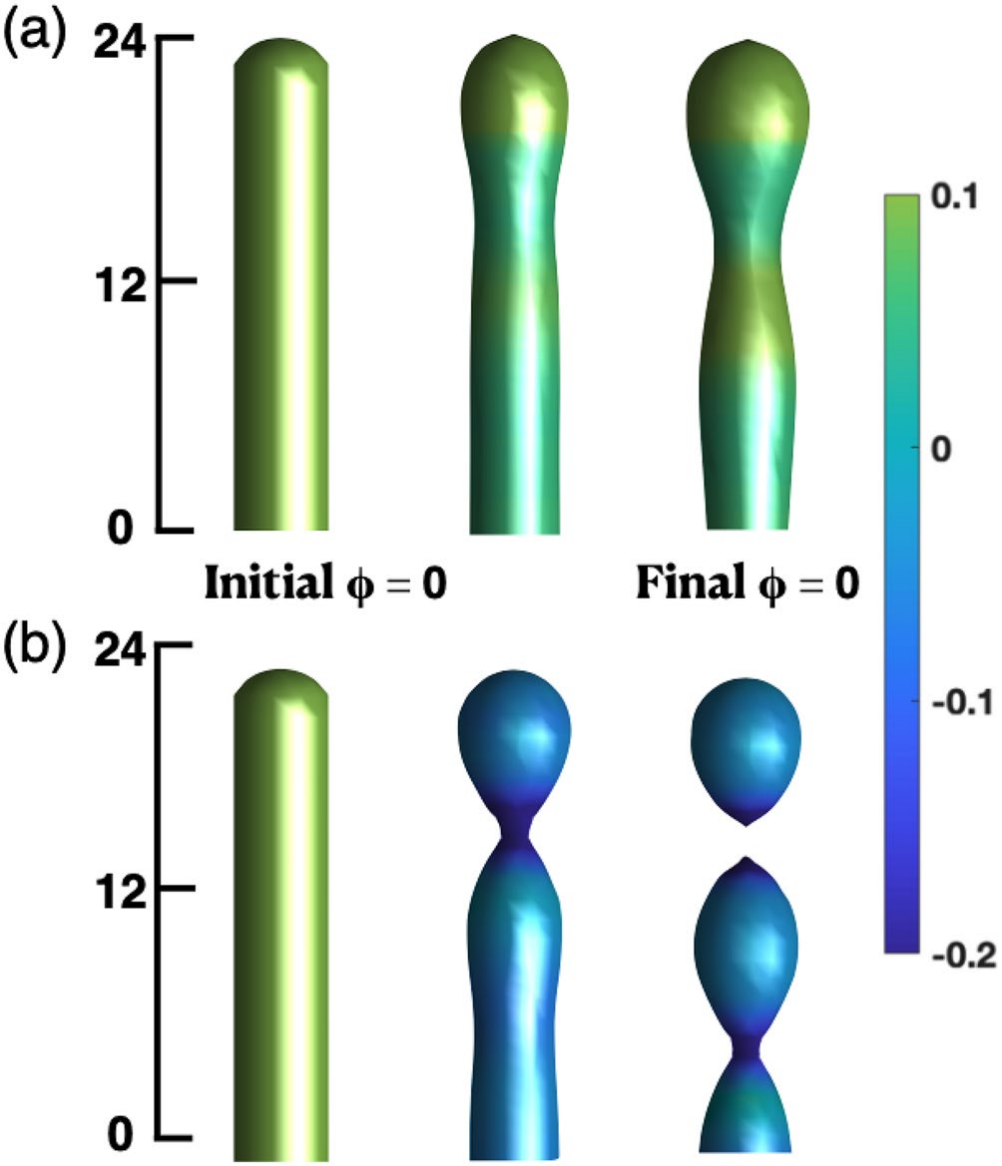
(**a**) Time evolution of the interface $\phi =0$ without considering Gaussian curvature ($\kappa '=0$). Snapshots of the membrane shape after two thousand, four million and ten millions of iterations (left to right). (**b**) Considering the Gaussian curvature contribution with $\kappa '=-10$. The snapshots are taken at the same time as in (**a**). The last snapshot depicts the exact moment when the vesicle breaks from the main membrane. The color code represents the local free energy in units of $\bar{\kappa }$. The initial condition for $\phi =0$ is taken after solving Eq. (9) 2000 iterations. The parameters used were: $R=5$ and $L=26$ in units of the domain grid and, $\varepsilon =1$, $\kappa =1$, $C_0 = -0.3$.

We start with the simplest phenomenon, the formation of two closed membranes from one, the Gaussian curvature controlling the fission of a single vesicle. The results are shown in Fig. 1 for the cases when (a) the Gaussian curvature term is not included in the free energy, and (b) solving the complete dynamical equation (9).

For the case when the Gaussian curvature is not considered ($\kappa '=0$ in Fig. 1a) a single neck forms, but there is no fission of the membrane. These results are consistent with previous works in which the Gaussian curvature is ignored^33^.

The addition of the Gaussian term in Eq. (9) makes the fission of the membrane energetically favorable and a vesicle is formed from the initial cylinder (Fig. 1b). We take $-\bar{\kappa }' \approx \bar{\kappa }$, as estimated in experiments and simulations alike^13,14^ and in the figure we indicated the local free energy in units of $\bar{\kappa }$. The size of this system only allows the cylinder to make a single vesicle. The dimensions of the vesicles can be predicted by a dispersion relation calculation shown below.

**Figure 2:**
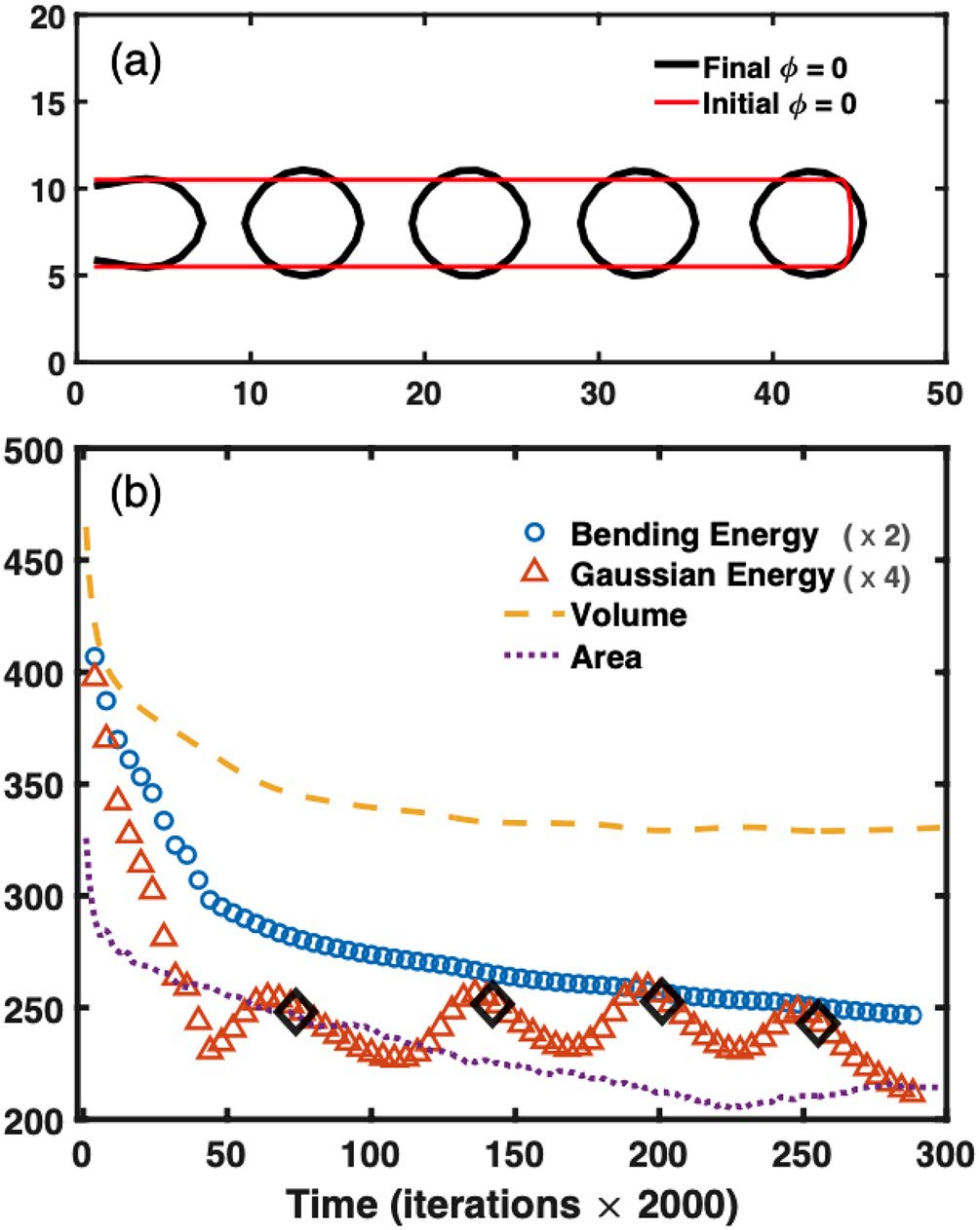
Time evolution of a longer membrane tube with Gaussian curvature. (**a**) Contour plots on the plane *x*, *z* of the initial (red) and final (black) $\phi =0$ configuration. (**b**) Time variations of the volume (yellow dashed line), the area (purple dotted line), the bending energy (open blue circles) and the Gaussian energy (open red triangles). The open black diamonds represent the times when the vesicle is formed. The parameters used were: $R=6$ and $L=45$, $\varepsilon =1$, $\kappa '=-10$ and $C_0=-0.5$.

For longer cylinders multiple fission events are possible. In Fig. 2, we show the results for a cylinder of length 45. We obtained a sequence of single fission events from the tip of the cylinder downwards. After $\sim 270,000$ time iterations, the cylinder splits into five separate closed vesicles. Shape changes start simultaneously through the entire tube, but the speed of pearling and fission is not equal. The tip of the cylinder changes shape faster and splits first. In Fig. 2a we show the initial and final configurations of $\phi =0$ for a longitudinal medial section of the tube. Although Helfrich–Landau bending energy was originally derived for nearly-flat membranes it has been successfully used to explain membranes with large curvatures^49^.

The time evolution of the volume, the area and the energy contributions is shown in Fig. 2b. The bending energy (open circles) and the Gaussian energy (open triangles) terms are drawn in absolute units of $\bar{\kappa }$. The changes in the Gaussian energy is half that of bending energy, which is in agreement with previous works^9^. The oscillating behavior is associated with the formation of vesicles in which the energy peaks are related to the necks narrowing and the following energy reduction is correlated with the formation of vesicles.

**Figure 3:**
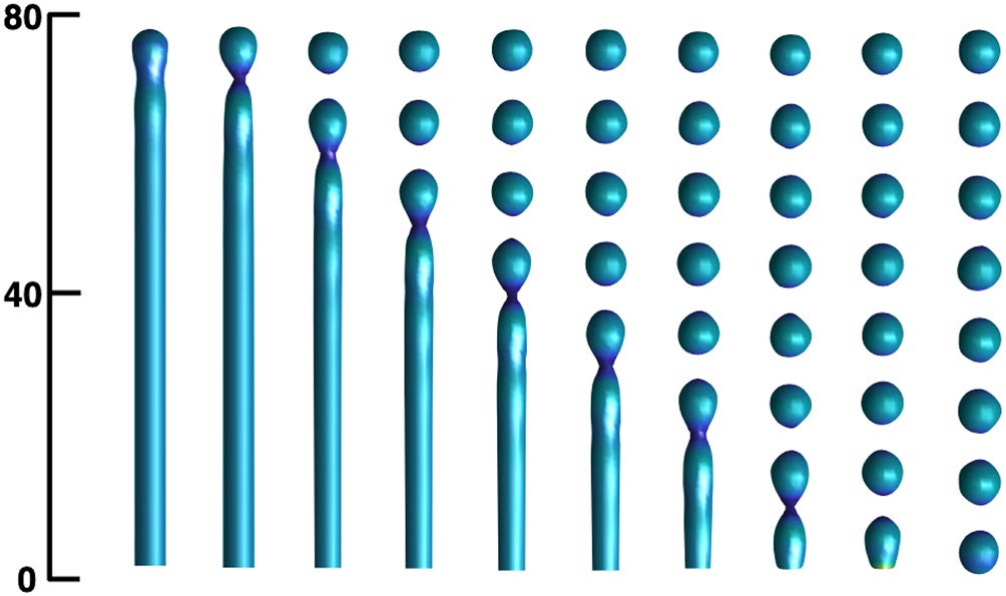
Snapshots of the evolution of the pearling instability in a long cylinder. Vesicles fission in sequence from the tip to the base. The parameters used were: $R=6$, $L=75$, $\varepsilon =1$, $\kappa =1$, $\kappa '=-10$ and $C_0=-0.5$.

Calculations on a longer cylinder are shown in Fig. 3. A pearling instability and an ordered fission is still observed. The onset of the pearling instability in Fig. 3 also appears on previous works^33^, but here the pearls fission due to the Gaussian curvature contribution. We conclude that Gaussian curvature controls the formation of many small vesicles from one big elongated vesicle as seen in the experimental results^15,16^.

All simulations were carried out having a reservoir of volume and area at the base of the cylinder. Therefore, global conservation of area and volume is not necessary. However, conservation of area and volume for individual vesicles should hold. As the vesicles fission from the main membrane, they lose contact with any reservoir of volume and area. Thus, from the moment they split, the vesicles must maintain their area and volume. In Fig. 2b it can be noticed that the area and volume vary slightly, although these values for the individual vesicles remain stable.

In order to analyse the stability of neck formation, it is possible to study the effects of small perturbations around the flat interface. These perturbations are taken as plane waves of the form $\phi = \phi _0e^{i{\mathbf {q\cdot x}} - \omega t}$, near $\phi = 0$, with small amplitudes, $\phi _0\ll 1$. Substituting these expressions into Eq. (9), and considering isotropic perturbations, one obtains the dispersion relation

11 $$\begin{aligned} \omega (q)&= 3q^2\kappa \big [(1 -2\varepsilon ^2C^2_0) +12\delta \varepsilon C_0\big ]-9q^4\kappa \varepsilon ^2 \big [ 2(1 + 4\delta \varepsilon C_0) \big ] -9q^4\kappa \sigma + 27q^6\kappa \varepsilon ^4. \end{aligned}$$

**Figure 4:**
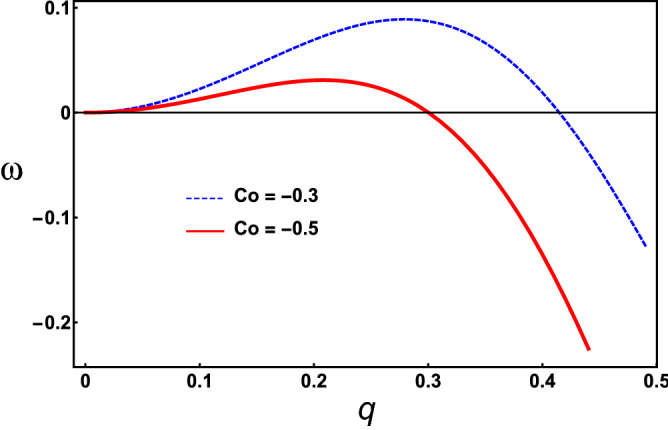
Dispersion relation with different values of spontaneous curvature. The parameter values are: $\kappa = 1$, $\varepsilon = 1$, $\delta = 0.001$ and $\sigma = 0.1$.

Here, $\delta$ represents terms of order ${\mathscr {O}}(\phi ^2)$. The detailed derivation of the dispersion relation is given in “Appendix B”. The main contribution to the instability comes from the first term in Eq. (11), in which the spontaneous curvature predominates. As the Gaussian curvature (due to $\kappa '$) is not present in Eq. (11), it does not alter the region of unstable wavelengths. The Gaussian curvature acts by causing topological changes of the membrane right in the sites where the instability occurs. The periodicity of the instability is determined by a critical length, $l_c = \pi /q_c$, where $q_c>0$ given by the point in which the unstable branch ceases to be positive $(\omega (q)=0)$. This quantity represents a scale for the formation of the necks. The dispersion relation for typical values of the parameters is depicted in Fig. 4. There, the corresponding values of the critical length in domain units are: $l_c \approx 7.6$ and $l_c \approx 10$ when $C_0 = -0.3$ and $C_0 = -0.5$, respectively. This is in agreement with the size (number) of vesicles formed in Figs. 1, 2, and 3 and $\varepsilon =1$ which is small, compared with the size of the system^50^. For instance, in Fig. 3 eight vesicles can be formed in a tube of length $L=75$ and a spontaneous curvature of $C_0=-0.5$, as the critical length is $l_c \approx 10$ in domain units for this case. Similar results are observed for shorter cylinders and the corresponding values of $C_0$ in Figs. 1 and 2.

**Figure 5:**
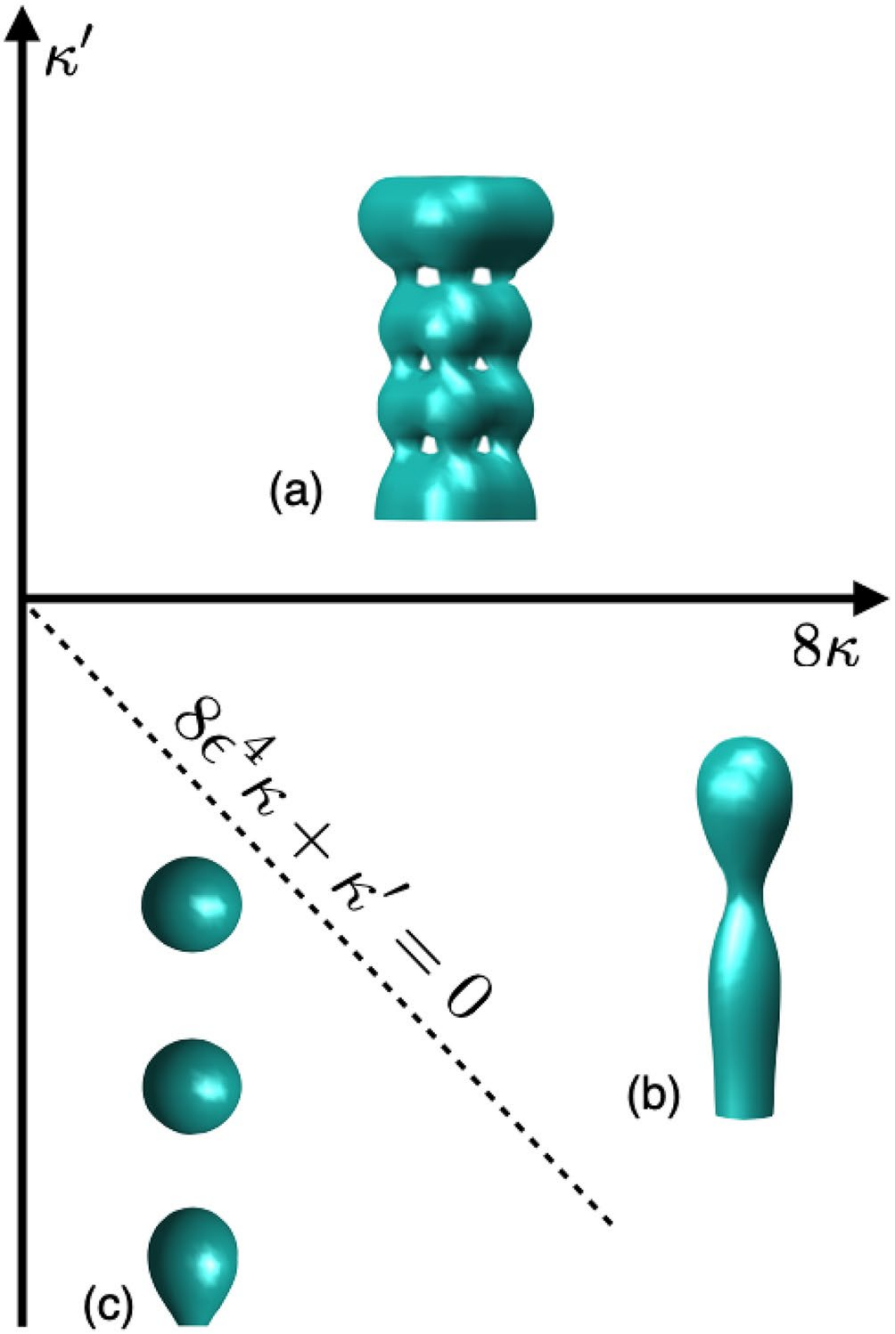
Membrane shape diagram for the ($\kappa$, $\kappa '$) landscape with $\varepsilon =1$. (**a**) Multiple self-connected membrane with positive $\kappa '$, (**b**) neck formation without fission for $\kappa ' > - 8\kappa$, and (**c**) vesicle formation with a magnitude of $\kappa ' < -8\kappa$.

Finally, we explore the regime in which the Gaussian bending modulus $\kappa '$ determines whether vesicle formation happens or not. The results in Fig. 5 show the equilibrium configurations for a single continuous membrane with tubular shape. For negative $\kappa '$ the transition from a single vesicle to to many small vesicles, occurs along the line where $8\varepsilon ^4\kappa + \kappa ' = 0$ (dotted line in Fig. 5). When $\kappa '$ is positive there is the formation of a multiple self-connected membrane, a continuous single membrane with multiple holes.

## Discussion

In previous works^29^ the Gaussian term has been worked out only in the case when the tube is stable, and pearled tubes are obtained, without pinching. Other works^51^ focus on the microstructure of the membrane, which is basically a liquid crystal film, and suggest that topological defects could produce fission. By contrast, our model is focus in the mesoscopic description of the fission process, which may include proteins that could influence the local curvature of the membrane.

In some experimental works^16^, pearling on tubes is obtained and after some time one observes fission of the pearls. In other experiments^44^ pearls occur in sequence, starting at the tip but without fission. It our numerical results vesicle pearling and fission occur in sequence, starting at the tip, it is possible that new experiments with the appropriate proteins exhibits this behavior.

Here we have studied the fission of membranes (vesicle formation) by extending the bending free energy model through the introduction of the Gaussian curvature. The Gaussian energy term provides the pearling instability the ability to fission from the initial membrane and maintain the stability of the system. With this model, multiple fission events can be obtained in a single computation.

The dynamics of vesicle formation was studied numerically and an ordered fission of the tube, from the tip to the base of the tube, was obtained. The number of formed vesicles depends on the dimensions of the tubular domain and the value of the spontaneous curvature. This number could be predicted using the dispersion relation obtained from a linear analysis of the model.

Topological changes were explored taking into account the two bending modules, giving us a membrane shape diagram. We corroborate the existence of multiple vesicles for negative $\kappa '$ for the values $\kappa ' < - 8\varepsilon ^4\kappa$. For Gaussian modulus $\kappa ' > - 8\varepsilon ^4\kappa$ no topological transition occurs. For positive $\kappa '$ the result is a multiple self-connected membrane.

To summarize, numerical calculations based on this model describe fission of membrane tubes into multiples vesicles. Pearling and vesicle fission occur in sequence from the tip of the tube to the base. Using the bending modulus and the Gaussian modulus we can obtain a membrane shape diagram, and we explored the different regimes. The model can represent systems where appropriate proteins are required for fission by means of the Gaussian energy.

## Appendix

### Appendix A: Calculation of the complete dynamic equation for $\phi$

Variations of ${\mathscr {F_{SC}}}$ in Eq. (8) are explicitly,

12 $$\begin{aligned} \frac{\delta \mathscr {F_{SC}}}{\delta \phi }=(3\phi ^2-1-2\phi \varepsilon C_0)\mu -\varepsilon \nabla ^2\mu + \sigma [\phi ]\nabla ^2\phi , \end{aligned}$$

where

13 $$\begin{aligned} \mu [\phi ]=(\phi -\varepsilon C_0)(\phi ^2-1)-\varepsilon ^2\nabla ^2\phi , \end{aligned}$$

is the chemical potential and $\sigma [\phi ]$ is the surface tension coefficient, which is implemented as a Lagrange multiplier that ensures local area conservation.

It is necessary to express Eq. (1) in terms of $\phi$ so we might be able to establish the variations of the energy due to the Gaussian curvature similar to Eq. (12). These variations must also be subject to diffusion so we can obtain the dynamic equation that dictates the evolution of the surface. Variations of the free energy due to the Gaussian curvature can be written as

14 $$\begin{aligned} \frac{\delta {\mathscr {F_G}}}{\delta \phi } = \frac{\partial \tilde{K}}{\partial \phi } - \partial _\gamma \cdot \frac{\partial \tilde{K}}{\partial (\partial _\gamma \phi )} + \partial _\gamma \partial _\gamma \cdot \frac{\partial \tilde{K}}{\partial (\partial _\gamma \partial _\gamma \phi )}. \end{aligned}$$

We write this last equation in terms of the curvature tensor $Q_{\alpha \beta }$, that is, in terms of the gradients of $\phi$, and then calculate each of the terms of Eq. (14) separately.

First, variations of $\tilde{K}$ with respect to $\phi$ can be written as:

15 $$\begin{aligned} \frac{\partial \tilde{K}}{\partial \phi } = \frac{2\sqrt{2}\varepsilon (\phi ^2+1)}{1-\phi ^2} \left[ (\partial _\alpha \phi )^2 Q_{\beta \beta } + (\partial _\beta \phi )^2 Q_{\alpha \alpha }\right] - \frac{2\sqrt{2}\varepsilon (\phi ^2+1)}{1-\phi ^2} \left[ 2 \partial _\alpha \phi \partial _\beta \phi Q_{\alpha \beta }\right] . \end{aligned}$$

The second term of Eq. (14) is

16 $$\begin{aligned} \begin{aligned} \partial _\gamma \cdot \frac{\partial \tilde{K}}{\partial (\partial _\gamma \phi )}&= \frac{4\sqrt{2}\varepsilon (1-3\phi ^2)}{1-\phi ^2} \left[ (\partial _\alpha \phi )^2 Q_{\beta \beta } + (\partial _\beta \phi )^2 Q_{\alpha \alpha }\right] -\frac{4\sqrt{2}\varepsilon (1-3\phi ^2)}{1-\phi ^2} \left[ 2 \partial _\alpha \phi \partial _\beta \phi Q_{\alpha \beta }\right] \\&\quad + 8\phi (1-\phi ^2)\left( Q_{\alpha \alpha }Q_{\beta \beta } - Q_{\alpha \beta }^2 \right) , \end{aligned} \end{aligned}$$

and the third term is:

17 $$\begin{aligned} \begin{aligned} \partial _\gamma \partial _\gamma \cdot \frac{\partial \tilde{K}}{\partial (\partial _\gamma \partial _\gamma \phi )}&= -4\phi (1-\phi ^2) \left[ Q_{\alpha \alpha }Q_{\beta \beta } - Q_{\alpha \beta }^2\right] + 4\sqrt{2}\varepsilon \phi ^2 \left[ (\partial _\alpha \phi )^2 Q_{\beta \beta } + (\partial _\beta \phi )^2 Q_{\alpha \alpha }\right] \\&\quad + 4\sqrt{2}\varepsilon \phi ^2 (-2 \partial _\alpha \phi \partial _\beta \phi Q_{\alpha \beta }). \end{aligned} \end{aligned}$$

By adding up Eqs. (15), (16) and (17), and grouping similar terms we have:

18 $$\begin{aligned} \begin{aligned} \frac{\delta{\mathscr {F_G}}}{\delta \phi }&= -12\phi (1-\phi ^2) \left[ Q_{\alpha \alpha }Q_{\beta \beta } - Q_{\alpha \beta }^2 \right] + \frac{2\sqrt{2}\varepsilon (9\phi ^2-1)}{1-\phi ^2} \left[ (\partial _\alpha \phi )^2 Q_{\beta \beta } + (\partial _\beta \phi )^2 Q_{\alpha \alpha }\right] \\&\quad - \frac{2\sqrt{2}\varepsilon (9\phi ^2-1)}{1-\phi ^2}\left[ 2\partial _\alpha \phi \partial _\beta \phi Q_{\alpha \beta } \right] . \end{aligned} \end{aligned}$$

It is possible to express the variations of the free energy due to the Gaussian curvature in terms of $\tilde{K}$ itself:

19 $$\begin{aligned} \frac{\delta \mathscr {F_G}}{\delta \phi } = \frac{(9\phi ^2-1)}{\phi ^2+1} \frac{\partial \tilde{K}}{\partial \phi } - \frac{12\phi }{1-\phi ^2} \tilde{K}. \end{aligned}$$

It is notable that the second term has a singularity when $\phi =\pm 1$ (that is, in the bulk) and it also approaches to zero near the interface $\phi =0$. Thus, the main contribution to the curvature energy of the surface comes from the first term of Eq. (19), which depends on the variation of the Gaussian curvature with respect to the order parameter $\phi$.

It is more appropriate to write the dynamical equations in terms of $\phi$. Thus, Eq. (19) is equivalent to

20 $$\begin{aligned} \begin{aligned} \frac{\delta \mathscr {F_G}}{\delta \phi }&= 2\varepsilon ^2\frac{-12\phi }{1-\phi ^2}\left[ \partial _{\alpha \alpha } \phi \partial _{\beta \beta } \phi - (\partial _{\alpha \beta } \phi )^2 \right] - 2\varepsilon ^2\frac{2(3\phi ^2+1)}{(1-\phi ^2)^2} \left[ \partial _{\alpha \alpha } \phi (\partial _\beta \phi )^2 + \partial _{\beta \beta } \phi (\partial _\alpha \phi )^2 \right] \\&\quad + 2\varepsilon ^2\frac{4(3\phi ^2+1)}{(1-\phi ^2)^2}\left[ \partial _{\alpha \beta } \phi \partial _{\alpha }\phi \partial _{\beta } \phi \right] . \end{aligned} \end{aligned}$$

The dynamic equation that dictates the evolution of the surface is

21 $$\begin{aligned} \begin{aligned} \frac{\partial \phi }{\partial t}&= \kappa \nabla ^2 \left( (3\phi ^2-1-2\phi \varepsilon C_0)\mu -\varepsilon \nabla ^2\mu + \sigma [\phi ]\nabla ^2\phi \right) -\kappa ' \nabla ^2 \left( \frac{12\phi }{1-\phi ^2} \left[ \partial _{\alpha \alpha } \phi \partial _{\beta \beta } \phi - (\partial _{\alpha \beta } \phi )^2 \right] \right) \\&\quad -\kappa '\nabla ^2 \left( \frac{2(3\phi ^2+1)}{(1-\phi ^2)^2} \left[ \partial _{\alpha \alpha } \phi (\partial _\beta \phi )^2 + \partial _{\beta \beta } \phi (\partial _\alpha \phi )^2 \right] \right) + \kappa '\nabla ^2 \left( \frac{4(3\phi ^2+1)}{(1-\phi ^2)^2} \left[ \partial _{\alpha \beta } \phi \partial _{\alpha }\phi \partial _{\beta } \phi \right] \right) , \end{aligned} \end{aligned}$$

which is defined only in terms of $\phi$ and its gradients and $\kappa ' = 3\sqrt{2}\varepsilon \bar{\kappa }'/4$.

### Appendix B: Dispersion relation

In order to analyse the stability of the membrane, one can study the effect of small perturbations of the flat interface. In particular, one considers that perturbations take the form of plane waves: $\phi = \phi _0e^{i\mathbf {q\cdot x}-\omega t}$, where $\mathbf {q}=$ $~q_{\alpha }$ and $\mathbf {x} =$ $~x_\alpha$ are the wave vector and Cartesian coordinates ($\alpha =1,2,3$), around the membrane $\phi = 0$. We assume that the amplitude $\phi _0 \ll 1$ is small.

Taking into account Eq. (9) of the main text and substituting the approximation in each term, one obtains

22 $$\begin{aligned} \begin{aligned} \nabla ^2\big ( (3\phi ^2 -1-2\phi \varepsilon C_0)\mu \big )&=-q^2_\alpha \phi \big [ (3\phi ^2-1-2\phi \varepsilon C_0)^2 +( 6\phi - 2\varepsilon C_0)(\phi - \varepsilon C_0)(\phi ^2 -1) \big ] \\&\quad - q^4_\alpha \varepsilon ^2\phi \big [(3\phi ^2-1-2\phi \varepsilon C_0)\\&\quad + \phi (6\phi -2\varepsilon C_0) \big ],\\ \end{aligned} \end{aligned}$$

since

23 $$\begin{aligned} \nabla ^2\mu = -q^2_\alpha \phi (3\phi ^2-1-2\phi \varepsilon C_0) - q^4_\alpha \varepsilon ^2\phi . \end{aligned}$$

The next term on the right hand side of Eq. (9) is,

24 $$\begin{aligned} \nabla (-\varepsilon ^2\nabla \mu )= -q^4_\alpha \varepsilon ^2\phi \big [(3\phi ^2-1-2\phi \varepsilon C_0)+\phi (6\phi -2\varepsilon C_0)\big ] -q^6_\alpha \varepsilon ^4\phi . \end{aligned}$$

On the other hand, one obtains,

25 $$\begin{aligned} F_{K_1} = F_{K_2} =0. \end{aligned}$$

The last equality is to be expected since in the plane wave approximation the functional associated with the Gaussian curvature is

26 $$\begin{aligned} \tilde{K} =\sum _{\alpha <\beta } (1-\phi ^2)^2 \left[ Q_{\alpha \alpha }Q_{\beta \beta }-Q_{\alpha \beta }^2 \right] =0. \end{aligned}$$

Now, if one substitutes Eqs. B1–B4 in Eq. (9), one obtains

27 $$\begin{aligned} \begin{aligned} \omega (q) \phi&= q^2_\alpha \kappa \phi \big [ (6\phi -2\varepsilon C_0)(\phi -\varepsilon C_0)(\phi ^2 -1)+ (3\phi ^2 - 1 -2\phi \varepsilon C_0)^2 \big ] +q^4_\alpha \kappa \varepsilon ^2\phi \big [ 2\phi (6\phi -2\varepsilon C_0) \\&\quad +2(3\phi ^2 - 1 -2\phi \varepsilon C_0)\big ]\\&\quad -q^4_\alpha \kappa \sigma \phi + q^6_\alpha \kappa \varepsilon ^4\phi .\\ \end{aligned} \end{aligned}$$

Assuming an isotropic perturbation $q_\alpha = q$ with small wave number, and using $\phi :=\delta$ for the terms of the order $\mathscr {O}(\phi ^2)$, one finally obtains the dispersion relation

28 $$\begin{aligned} \omega (q) = 3q^2\kappa \big [(1 -2\varepsilon ^2 C^2_0) +12\delta \varepsilon C_0\big ]-9q^4\kappa \varepsilon ^2 \big [ 2(1 +4\delta \varepsilon C_0) \big ] -9q^4\kappa \sigma + 27q^6\kappa \varepsilon ^4. \end{aligned}$$

From this result, one observes that the spontaneous curvature is responsible for the onset of the instability and that the mean and Gaussian curvature do not affect the region of the unstable wavelengths.
